# Ethyl 4-hy­droxy-6-(4-hy­droxy­phen­yl)-4-trifluoro­methyl-2-sulfanyl­idene-1,3-diazinane-5-carboxyl­ate ethanol monosolvate

**DOI:** 10.1107/S1600536811015376

**Published:** 2011-04-29

**Authors:** Feng-Ling Yang, Wen-Jun Fa

**Affiliations:** a College of Chemistry and Chemical Engineering, Pingdingshan University, Pingdingshan, Henan Province 467000, People’s Republic of China; b Institute of Surface Micro and Nano Materials, Xuchang University, Xuchang, Henan Province 461000, People’s Republic of China

## Abstract

The title compound, C_14_H_15_F_3_N_2_O_4_S·C_2_H_5_OH, was prepared by reaction of 4-hy­droxy­benzaldehyde, ethyl 4,4,4-trifluoro-3-oxobutano­ate and thio­urea. The hexa­hydro­pyrimidine ring adopts a half-chair conformation, the mean plane formed by the ring atoms excluding the C atom bonded to the eth­oxy­carbonyl group has an r.m.s. deviation of 0.0333 Å, and the dihedral angle between this plane and the benzene ring is 56.76 (5)°. The mol­ecular conformation is stabilized by an intra­molecular O—H⋯O hydrogen bond, generating an *S*(6) ring. The crystal structure is stabilized by inter­molecular O—H⋯O, O—H⋯S, N—H⋯O and N—H⋯S hydrogen bonds. The ethyl group of the ester unit is disordered over two positions, with an occupancy ratio of 0.757 (10):0.243 (10).

## Related literature

For the bioactivity of dihydro­pyrimidines, see: Brier *et al.* (2004 ▶); Cochran *et al.* (2005 ▶); Moran *et al.* (2007 ▶); Zorkun *et al.* (2006 ▶). For the bioactivity of organofluorine compounds, see: Hermann *et al.* (2003 ▶); Ulrich (2004 ▶). For a related structure, see: Song *et al.* (2010 ▶).
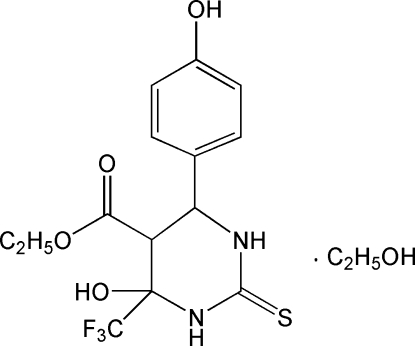


## Experimental

### 

#### Crystal data

C_14_H_15_F_3_N_2_O_4_S·C_2_H_6_O
*M* *_r_* = 410.41
Monoclinic, 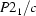

*a* = 14.7204 (14) Å
*b* = 9.9772 (12) Å
*c* = 14.7357 (15) Å
β = 119.716 (11)°
*V* = 1879.6 (3) Å^3^
*Z* = 4
Mo *K*α radiation
μ = 0.23 mm^−1^
*T* = 113 K
0.20 × 0.16 × 0.10 mm

#### Data collection

Rigaku Saturn CCD area-detector diffractometer
Absorption correction: multi-scan (*CrystalClear*; Rigaku, 2009 ▶) *T*_min_ = 0.955, *T*_max_ = 0.977
23471 measured reflections
4486 independent reflections
3815 reflections with *I* > 2σ(*I*)
*R*_int_ = 0.047

#### Refinement

*R*[*F* ^2^ > 2σ(*F* ^2^)] = 0.036
*wR*(*F* ^2^) = 0.092
*S* = 1.03
4486 reflections
280 parameters
24 restraints
H atoms treated by a mixture of independent and constrained refinement
Δρ_max_ = 0.47 e Å^−3^
Δρ_min_ = −0.27 e Å^−3^

### 

Data collection: *CrystalClear* (Rigaku, 2009 ▶); cell refinement: *CrystalClear*; data reduction: *CrystalClear*; program(s) used to solve structure: *SHELXS97* (Sheldrick, 2008 ▶); program(s) used to refine structure: *SHELXL97* (Sheldrick, 2008 ▶); molecular graphics: *SHELXTL* (Sheldrick, 2008 ▶); software used to prepare material for publication: *SHELXL97*.

## Supplementary Material

Crystal structure: contains datablocks global, I. DOI: 10.1107/S1600536811015376/om2424sup1.cif

Structure factors: contains datablocks I. DOI: 10.1107/S1600536811015376/om2424Isup2.hkl

Supplementary material file. DOI: 10.1107/S1600536811015376/om2424Isup3.cml

Additional supplementary materials:  crystallographic information; 3D view; checkCIF report

## Figures and Tables

**Table 1:** Hydrogen-bond geometry (Å, °)

*D*—H⋯*A*	*D*—H	H⋯*A*	*D*⋯*A*	*D*—H⋯*A*
O1—H1⋯O2	0.853 (19)	1.987 (19)	2.7383 (13)	146.3 (17)
O1—H1⋯S1^i^	0.853 (19)	3.029 (19)	3.4858 (11)	115.8 (15)
O4—H4⋯O5^ii^	0.835 (18)	1.873 (19)	2.7066 (14)	175.6 (19)
O5—H5⋯S1^iii^	0.84	2.36	3.1970 (11)	175
N1—H1*A*⋯S1^iii^	0.855 (17)	2.583 (18)	3.4307 (12)	171.5 (14)
N2—H2*A*⋯O1^iv^	0.814 (14)	2.368 (15)	3.1701 (15)	168.7 (14)

Symmetry codes: (i) 
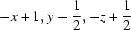
; (ii) 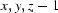; (iii) 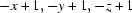; (iv) 
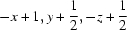
.

**Fig. 1.**
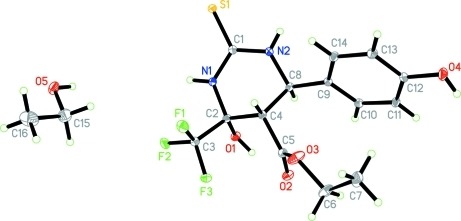
Molecular configuration and atom numbering scheme with displacement ellipsoids drawn at the 30% probability level.

## supplementary crystallographic information

### Comment

Dihydropyrimidine (DHPM) derivatives can be used as potential calcium channel blockers (Zorkun *et al.*, 2006), inhibitors of mitotic kinesin Eg5 for treating cancer (Cochran *et al.*, 2005; Brier *et al.*, 2004) and as TRPA1 modulators for treating pain (Moran *et al.*, 2007). In addition, compounds that contain fluorine have special bioactivity, *e.g.* flumioxazin is a widely used herbicide (Hermann *et al.*, 2003; Ulrich, 2004). This led us to focus our attention on the synthesis and bioactivity of these important fused perfluoroalkylated heterocyclic compounds. During the synthesis of DHPM derivatives, the title compound, an intermediate C_14_H_15_F_3_N_2_O_4_S.C_2_H_5_OH was isolated and the structure confirmed by X-ray diffraction.

In the structure of the title molecule, the 1,3-diazinane ring adopts a half-chair conformation, the mean plane formed by the ring atoms excluding the C atom bonded to the ethoxy carbonyl group has an r.m.s. deviation of 0.0333 Å, the dihedral angle between the mean plane and benzene ring is 56.76 (5)°. The molecular conformation is stabilized by intramolecular O—H···O hydrogen bond, generating an S(6) ring. The crystal structure is stabilized by intermolecular hydrogen bonds (O—H···O, O—H···S, N—H···O and N—H···S). The ethyl group of the ester unit is disordered over two positions, with a site-occupancy ratio of 0.757 (10):0.243 (10). For a crystal structure related to the title compound, see: Song *et al.*, 2010.

### Experimental

The title compound was synthesized by refluxing for 3 h a stirred solution of 4-hydroxybenzaldehyde (2.45 g, 20 mmol), ethyl 4,4,4-trifluoro-3-oxobutanoate (4.42 g, 24 mmol) and thiourea (2.28 g, 30 mmol) in 20 ml of anhydrous ethanol. The reaction was catalyzed by sulfamic acid (0.6 g). The solvent was evaporated *in vacuo* and the residue was washed with water. The title compound was recrystallized by slow evaporation of a 50% aqueous ethanol solution.

### Refinement

H atoms involved in hydrogen-bonding inetractions were located by difference Fourier methods and their positional and isotropic displacement parameters were refined. Other H atoms were placed in calculated positions, with C—H(aromatic) = 0.95 Å and C—H(aliphatic) = 0.98 Å or 0.99 Å, and treated as riding, with *U*_iso_(H) = 1.2*U*eq(C).

### Crystal data

**Table:**

C_14_H_15_F_3_N_2_O_4_S·C_2_H_6_O	*F*(000) = 856
*M**_r_* = 410.41	*D*_x_ = 1.450 Mg m^−^^3^
Monoclinic, *P*2_1_/*c*	Mo *K*α radiation, λ = 0.71073 Å
*a* = 14.7204 (14) Å	Cell parameters from 6768 reflections
*b* = 9.9772 (12) Å	θ = 2.0–27.9°
*c* = 14.7357 (15) Å	µ = 0.23 mm^−^^1^
β = 119.716 (11)°	*T* = 113 K
*V* = 1879.6 (3) Å^3^	Prism, colorless
*Z* = 4	0.20 × 0.16 × 0.10 mm

### Data collection

**Table:**

Rigaku Saturn CCD area-detector diffractometer	4486 independent reflections
Radiation source: rotating anode	3815 reflections with *I* > 2σ(*I*)
multilayer	*R*_int_ = 0.047
Detector resolution: 14.63 pixels mm^-1^	θ_max_ = 27.9°, θ_min_ = 2.6°
ω and φ scans	*h* = −18→19
Absorption correction: multi-scan (*CrystalClear*; Rigaku, 2009)	*k* = −13→13
*T*_min_ = 0.955, *T*_max_ = 0.977	*l* = −19→19
23471 measured reflections	

### Refinement

**Table:**

Refinement on *F*^2^	Primary atom site location: structure-invariant direct methods
Least-squares matrix: full	Secondary atom site location: difference Fourier map
*R*[*F*^2^ > 2σ(*F*^2^)] = 0.036	Hydrogen site location: inferred from neighbouring sites
*wR*(*F*^2^) = 0.092	H atoms treated by a mixture of independent and constrained refinement
*S* = 1.03	*w* = 1/[σ^2^(*F*_o_^2^) + (0.0506*P*)^2^] where *P* = (*F*_o_^2^ + 2*F*_c_^2^)/3
4486 reflections	(Δ/σ)_max_ = 0.004
280 parameters	Δρ_max_ = 0.47 e Å^−^^3^
24 restraints	Δρ_min_ = −0.27 e Å^−^^3^

### Special details

**Table:**

Geometry. All e.s.d.'s (except the e.s.d. in the dihedral angle between two l.s. planes) are estimated using the full covariance matrix. The cell e.s.d.'s are taken into account individually in the estimation of e.s.d.'s in distances, angles and torsion angles; correlations between e.s.d.'s in cell parameters are only used when they are defined by crystal symmetry. An approximate (isotropic) treatment of cell e.s.d.'s is used for estimating e.s.d.'s involving l.s. planes.
Refinement. Refinement of *F*^2^ against ALL reflections. The weighted *R*-factor *wR* and goodness of fit *S* are based on *F*^2^, conventional *R*-factors *R* are based on *F*, with *F* set to zero for negative *F*^2^. The threshold expression of *F*^2^ > σ(*F*^2^) is used only for calculating *R*-factors(gt) *etc*. and is not relevant to the choice of reflections for refinement. *R*-factors based on *F*^2^ are statistically about twice as large as those based on *F*, and *R*- factors based on ALL data will be even larger.

### Fractional atomic coordinates and isotropic or equivalent isotropic displacement parameters (Å^2^)

**Table:**

	*x*	*y*	*z*	*U*_iso_*/*U*_eq_	Occ. (<1)
S1	0.42068 (2)	0.59475 (3)	0.35546 (2)	0.01745 (10)	
F1	0.79908 (6)	0.50693 (8)	0.56642 (6)	0.0289 (2)	
F2	0.74838 (6)	0.31639 (8)	0.59449 (6)	0.0272 (2)	
F3	0.85429 (6)	0.32318 (9)	0.53355 (6)	0.0293 (2)	
O1	0.65426 (7)	0.24640 (9)	0.38459 (7)	0.0190 (2)	
H1	0.6926 (15)	0.2229 (18)	0.3589 (15)	0.056 (6)*	
O2	0.79221 (7)	0.28215 (9)	0.31283 (7)	0.0234 (2)	
O3	0.85300 (8)	0.49263 (10)	0.32835 (9)	0.0356 (3)	
O4	0.68310 (8)	0.73637 (10)	−0.05667 (8)	0.0264 (2)	
H4	0.7053 (15)	0.6801 (19)	−0.0829 (14)	0.048 (6)*	
N1	0.58962 (8)	0.44198 (11)	0.41833 (8)	0.0161 (2)	
N2	0.52429 (9)	0.53998 (11)	0.25624 (8)	0.0171 (2)	
C1	0.51772 (9)	0.52182 (12)	0.34218 (9)	0.0154 (3)	
C2	0.67753 (10)	0.38150 (12)	0.41637 (10)	0.0156 (3)	
C3	0.77017 (10)	0.38279 (13)	0.52882 (10)	0.0199 (3)	
C4	0.70305 (9)	0.46428 (12)	0.34352 (9)	0.0153 (3)	
H4A	0.7255	0.5564	0.3730	0.018*	
C5	0.78765 (10)	0.40048 (13)	0.32782 (10)	0.0183 (3)	
C6	0.9245 (3)	0.4501 (3)	0.2899 (4)	0.0349 (8)	0.757 (10)
H6A	0.8925	0.3759	0.2391	0.042*	0.757 (10)
H6B	0.9914	0.4179	0.3490	0.042*	0.757 (10)
C7	0.9440 (2)	0.5681 (4)	0.2384 (2)	0.0371 (9)	0.757 (10)
H7A	0.9920	0.5418	0.2134	0.056*	0.757 (10)
H7B	0.9751	0.6413	0.2890	0.056*	0.757 (10)
H7C	0.8777	0.5981	0.1791	0.056*	0.757 (10)
C6'	0.9536 (7)	0.4377 (10)	0.3420 (11)	0.034 (2)	0.243 (10)
H6'A	0.9568	0.3387	0.3462	0.041*	0.243 (10)
H6'B	1.0158	0.4770	0.4028	0.041*	0.243 (10)
C7'	0.9382 (8)	0.4894 (18)	0.2394 (9)	0.056 (3)	0.243 (10)
H7'A	0.9987	0.4651	0.2320	0.083*	0.243 (10)
H7'B	0.9309	0.5872	0.2373	0.083*	0.243 (10)
H7'C	0.8748	0.4497	0.1820	0.083*	0.243 (10)
C8	0.60150 (10)	0.47359 (12)	0.23647 (9)	0.0157 (3)	
H8A	0.5765	0.3807	0.2107	0.019*	
C9	0.61729 (10)	0.54638 (12)	0.15579 (9)	0.0158 (3)	
C10	0.63212 (10)	0.47113 (13)	0.08457 (9)	0.0171 (3)	
H10A	0.6260	0.3763	0.0842	0.021*	
C11	0.65560 (10)	0.53228 (13)	0.01436 (10)	0.0179 (3)	
H11A	0.6675	0.4794	−0.0323	0.021*	
C12	0.66173 (10)	0.67134 (13)	0.01224 (10)	0.0179 (3)	
C13	0.64511 (11)	0.74801 (13)	0.08177 (10)	0.0215 (3)	
H13A	0.6479	0.8430	0.0799	0.026*	
C14	0.62449 (11)	0.68552 (13)	0.15354 (10)	0.0194 (3)	
H14A	0.6151	0.7382	0.2020	0.023*	
O5	0.74901 (7)	0.56068 (11)	0.84823 (8)	0.0276 (2)	
H5	0.7076	0.5158	0.7956	0.041*	
C15	0.84625 (12)	0.57515 (17)	0.84999 (13)	0.0359 (4)	
H15A	0.8351	0.6201	0.7854	0.043*	
H15B	0.8774	0.4859	0.8539	0.043*	
C16	0.91849 (13)	0.65741 (19)	0.94378 (15)	0.0484 (5)	
H16A	0.9857	0.6682	0.9460	0.073*	
H16B	0.9295	0.6120	1.0074	0.073*	
H16C	0.8873	0.7457	0.9391	0.073*	
H1A	0.5812 (12)	0.4270 (16)	0.4708 (13)	0.037 (5)*	
H2A	0.4826 (11)	0.5934 (14)	0.2148 (11)	0.018 (4)*	

### Atomic displacement parameters (Å^2^)

**Table:**

	*U*^11^	*U*^22^	*U*^33^	*U*^12^	*U*^13^	*U*^23^
S1	0.01884 (18)	0.01825 (17)	0.01816 (17)	0.00402 (12)	0.01138 (14)	0.00283 (12)
F1	0.0287 (5)	0.0258 (4)	0.0222 (4)	−0.0028 (4)	0.0051 (4)	−0.0062 (3)
F2	0.0265 (5)	0.0362 (5)	0.0191 (4)	0.0057 (4)	0.0116 (4)	0.0094 (3)
F3	0.0191 (4)	0.0438 (5)	0.0235 (4)	0.0123 (4)	0.0093 (4)	0.0032 (4)
O1	0.0237 (5)	0.0137 (5)	0.0235 (5)	−0.0001 (4)	0.0147 (4)	−0.0010 (4)
O2	0.0242 (5)	0.0206 (5)	0.0291 (5)	0.0010 (4)	0.0160 (4)	−0.0031 (4)
O3	0.0276 (6)	0.0282 (6)	0.0643 (8)	−0.0086 (5)	0.0330 (6)	−0.0118 (5)
O4	0.0427 (6)	0.0200 (5)	0.0283 (5)	0.0029 (4)	0.0267 (5)	0.0046 (4)
N1	0.0176 (6)	0.0183 (5)	0.0151 (5)	0.0028 (4)	0.0100 (5)	0.0027 (4)
N2	0.0167 (6)	0.0204 (6)	0.0145 (5)	0.0046 (5)	0.0080 (5)	0.0033 (4)
C1	0.0169 (6)	0.0133 (6)	0.0158 (6)	−0.0025 (5)	0.0080 (5)	−0.0010 (5)
C2	0.0159 (6)	0.0148 (6)	0.0165 (6)	0.0006 (5)	0.0084 (5)	0.0000 (5)
C3	0.0199 (7)	0.0216 (7)	0.0195 (6)	0.0037 (5)	0.0108 (6)	0.0014 (5)
C4	0.0167 (6)	0.0143 (6)	0.0161 (6)	−0.0005 (5)	0.0090 (5)	−0.0008 (5)
C5	0.0145 (6)	0.0229 (7)	0.0159 (6)	−0.0009 (5)	0.0063 (5)	−0.0011 (5)
C6	0.0241 (17)	0.0424 (15)	0.050 (2)	−0.0031 (12)	0.0270 (17)	−0.0049 (16)
C7	0.0250 (12)	0.062 (2)	0.0300 (12)	−0.0070 (13)	0.0177 (10)	0.0034 (13)
C6'	0.012 (4)	0.041 (4)	0.050 (6)	0.001 (3)	0.015 (4)	0.002 (4)
C7'	0.039 (5)	0.081 (8)	0.058 (5)	−0.005 (5)	0.033 (4)	0.002 (5)
C8	0.0175 (6)	0.0158 (6)	0.0154 (6)	−0.0005 (5)	0.0095 (5)	−0.0012 (5)
C9	0.0155 (6)	0.0167 (6)	0.0147 (6)	0.0008 (5)	0.0072 (5)	0.0009 (5)
C10	0.0196 (7)	0.0142 (6)	0.0179 (6)	−0.0006 (5)	0.0096 (6)	−0.0004 (5)
C11	0.0209 (7)	0.0184 (6)	0.0160 (6)	0.0000 (5)	0.0105 (6)	−0.0023 (5)
C12	0.0193 (7)	0.0190 (7)	0.0173 (6)	0.0018 (5)	0.0106 (6)	0.0034 (5)
C13	0.0292 (8)	0.0137 (6)	0.0251 (7)	0.0012 (5)	0.0161 (6)	0.0002 (5)
C14	0.0253 (7)	0.0172 (6)	0.0196 (6)	0.0010 (5)	0.0140 (6)	−0.0018 (5)
O5	0.0236 (5)	0.0372 (6)	0.0255 (5)	−0.0044 (4)	0.0149 (5)	−0.0081 (4)
C15	0.0264 (8)	0.0492 (10)	0.0368 (9)	−0.0002 (7)	0.0193 (8)	0.0010 (8)
C16	0.0275 (9)	0.0468 (11)	0.0586 (12)	−0.0065 (8)	0.0120 (9)	−0.0034 (9)

### Geometric parameters (Å, °)

**Table:**

S1—C1	1.6987 (13)	C7—H7C	0.9800
F1—C3	1.3373 (15)	C6'—C7'	1.504 (14)
F2—C3	1.3364 (15)	C6'—H6'A	0.9900
F3—C3	1.3440 (15)	C6'—H6'B	0.9900
O1—C2	1.4123 (15)	C7'—H7'A	0.9800
O1—H1	0.853 (19)	C7'—H7'B	0.9800
O2—C5	1.2090 (15)	C7'—H7'C	0.9800
O3—C5	1.3279 (16)	C8—C9	1.5063 (17)
O3—C6	1.483 (3)	C8—H8A	1.0000
O3—C6'	1.497 (9)	C9—C10	1.3927 (17)
O4—C12	1.3666 (15)	C9—C14	1.3939 (17)
O4—H4	0.835 (18)	C10—C11	1.3844 (17)
N1—C1	1.3551 (16)	C10—H10A	0.9500
N1—C2	1.4410 (16)	C11—C12	1.3917 (18)
N1—H1A	0.855 (17)	C11—H11A	0.9500
N2—C1	1.3294 (16)	C12—C13	1.3940 (18)
N2—C8	1.4640 (16)	C13—C14	1.3849 (18)
N2—H2A	0.814 (14)	C13—H13A	0.9500
C2—C3	1.5380 (18)	C14—H14A	0.9500
C2—C4	1.5413 (17)	O5—C15	1.4261 (16)
C4—C5	1.5149 (17)	O5—H5	0.8400
C4—C8	1.5472 (17)	C15—C16	1.503 (2)
C4—H4A	1.0000	C15—H15A	0.9900
C6—C7	1.504 (4)	C15—H15B	0.9900
C6—H6A	0.9900	C16—H16A	0.9800
C6—H6B	0.9900	C16—H16B	0.9800
C7—H7A	0.9800	C16—H16C	0.9800
C7—H7B	0.9800		
			
C2—O1—H1	107.7 (13)	O3—C6'—H6'A	112.7
C5—O3—C6	116.66 (15)	C7'—C6'—H6'A	112.7
C5—O3—C6'	114.4 (4)	O3—C6'—H6'B	112.7
C6—O3—C6'	26.4 (4)	C7'—C6'—H6'B	112.7
C12—O4—H4	108.2 (13)	H6'A—C6'—H6'B	110.2
C1—N1—C2	124.76 (11)	C6'—C7'—H7'A	109.5
C1—N1—H1A	116.6 (11)	C6'—C7'—H7'B	109.5
C2—N1—H1A	118.6 (11)	H7'A—C7'—H7'B	109.5
C1—N2—C8	123.93 (11)	C6'—C7'—H7'C	109.5
C1—N2—H2A	114.8 (10)	H7'A—C7'—H7'C	109.5
C8—N2—H2A	121.3 (10)	H7'B—C7'—H7'C	109.5
N2—C1—N1	118.24 (11)	N2—C8—C9	112.19 (10)
N2—C1—S1	120.92 (10)	N2—C8—C4	106.10 (10)
N1—C1—S1	120.84 (9)	C9—C8—C4	112.61 (10)
O1—C2—N1	109.49 (10)	N2—C8—H8A	108.6
O1—C2—C3	107.76 (10)	C9—C8—H8A	108.6
N1—C2—C3	107.48 (10)	C4—C8—H8A	108.6
O1—C2—C4	112.54 (10)	C10—C9—C14	118.48 (12)
N1—C2—C4	108.72 (10)	C10—C9—C8	118.55 (11)
C3—C2—C4	110.72 (10)	C14—C9—C8	122.83 (11)
F2—C3—F1	107.49 (10)	C11—C10—C9	121.06 (12)
F2—C3—F3	106.79 (10)	C11—C10—H10A	119.5
F1—C3—F3	107.01 (11)	C9—C10—H10A	119.5
F2—C3—C2	111.90 (11)	C10—C11—C12	119.94 (12)
F1—C3—C2	112.59 (10)	C10—C11—H11A	120.0
F3—C3—C2	110.75 (10)	C12—C11—H11A	120.0
C5—C4—C2	112.57 (10)	O4—C12—C11	122.09 (12)
C5—C4—C8	108.75 (10)	O4—C12—C13	118.32 (12)
C2—C4—C8	107.24 (10)	C11—C12—C13	119.58 (12)
C5—C4—H4A	109.4	C14—C13—C12	119.94 (12)
C2—C4—H4A	109.4	C14—C13—H13A	120.0
C8—C4—H4A	109.4	C12—C13—H13A	120.0
O2—C5—O3	124.85 (12)	C13—C14—C9	120.96 (12)
O2—C5—C4	124.23 (12)	C13—C14—H14A	119.5
O3—C5—C4	110.86 (11)	C9—C14—H14A	119.5
O3—C6—C7	108.6 (2)	C15—O5—H5	109.5
O3—C6—H6A	110.0	O5—C15—C16	108.56 (13)
C7—C6—H6A	110.0	O5—C15—H15A	110.0
O3—C6—H6B	110.0	C16—C15—H15A	110.0
C7—C6—H6B	110.0	O5—C15—H15B	110.0
H6A—C6—H6B	108.4	C16—C15—H15B	110.0
C6—C7—H7A	109.5	H15A—C15—H15B	108.4
C6—C7—H7B	109.5	C15—C16—H16A	109.5
H7A—C7—H7B	109.5	C15—C16—H16B	109.5
C6—C7—H7C	109.5	H16A—C16—H16B	109.5
H7A—C7—H7C	109.5	C15—C16—H16C	109.5
H7B—C7—H7C	109.5	H16A—C16—H16C	109.5
O3—C6'—C7'	95.3 (7)	H16B—C16—H16C	109.5
			
C8—N2—C1—N1	3.16 (18)	C8—C4—C5—O2	−75.56 (15)
C8—N2—C1—S1	−175.89 (9)	C2—C4—C5—O3	−139.51 (11)
C2—N1—C1—N2	4.26 (18)	C8—C4—C5—O3	101.80 (12)
C2—N1—C1—S1	−176.69 (9)	C5—O3—C6—C7	147.6 (2)
C1—N1—C2—O1	−100.03 (14)	C6'—O3—C6—C7	−120.7 (11)
C1—N1—C2—C3	143.19 (12)	C5—O3—C6'—C7'	119.4 (7)
C1—N1—C2—C4	23.29 (16)	C6—O3—C6'—C7'	18.2 (10)
O1—C2—C3—F2	−59.19 (13)	C1—N2—C8—C9	−159.43 (11)
N1—C2—C3—F2	58.73 (13)	C1—N2—C8—C4	−36.07 (16)
C4—C2—C3—F2	177.35 (10)	C5—C4—C8—N2	−178.07 (10)
O1—C2—C3—F1	179.61 (10)	C2—C4—C8—N2	59.94 (12)
N1—C2—C3—F1	−62.47 (13)	C5—C4—C8—C9	−54.97 (13)
C4—C2—C3—F1	56.15 (14)	C2—C4—C8—C9	−176.96 (10)
O1—C2—C3—F3	59.84 (13)	N2—C8—C9—C10	−141.94 (12)
N1—C2—C3—F3	177.76 (10)	C4—C8—C9—C10	98.43 (13)
C4—C2—C3—F3	−63.63 (14)	N2—C8—C9—C14	42.35 (16)
O1—C2—C4—C5	−52.75 (14)	C4—C8—C9—C14	−77.28 (15)
N1—C2—C4—C5	−174.22 (10)	C14—C9—C10—C11	1.30 (18)
C3—C2—C4—C5	67.91 (13)	C8—C9—C10—C11	−174.60 (11)
O1—C2—C4—C8	66.82 (13)	C9—C10—C11—C12	−1.85 (19)
N1—C2—C4—C8	−54.65 (13)	C10—C11—C12—O4	−178.99 (12)
C3—C2—C4—C8	−172.52 (10)	C10—C11—C12—C13	0.62 (19)
C6—O3—C5—O2	11.0 (3)	O4—C12—C13—C14	−179.24 (12)
C6'—O3—C5—O2	−18.2 (6)	C11—C12—C13—C14	1.14 (19)
C6—O3—C5—C4	−166.3 (2)	C12—C13—C14—C9	−1.7 (2)
C6'—O3—C5—C4	164.5 (6)	C10—C9—C14—C13	0.48 (19)
C2—C4—C5—O2	43.13 (17)	C8—C9—C14—C13	176.20 (12)

### Hydrogen-bond geometry (Å, °)

**Table:**

*D*—H···*A*	*D*—H	H···*A*	*D*···*A*	*D*—H···*A*
O1—H1···O2	0.853 (19)	1.987 (19)	2.7383 (13)	146.3 (17)
O1—H1···S1^i^	0.853 (19)	3.029 (19)	3.4858 (11)	115.8 (15)
O4—H4···O5^ii^	0.835 (18)	1.873 (19)	2.7066 (14)	175.6 (19)
O5—H5···S1^iii^	0.84	2.36	3.1970 (11)	175
N1—H1A···S1^iii^	0.855 (17)	2.583 (18)	3.4307 (12)	171.5 (14)
N2—H2A···O1^iv^	0.814 (14)	2.368 (15)	3.1701 (15)	168.7 (14)

Symmetry codes: (i) −*x*+1, *y*−1/2, −*z*+1/2; (ii) *x*, *y*, *z*−1; (iii) −*x*+1, −*y*+1, −*z*+1; (iv) −*x*+1, *y*+1/2, −*z*+1/2.
